# Generation and Evaluation of a Multi-Epitope Vaccine Against *Acinetobacter baumannii*, a Nosocomial Bacterial Pathogen

**DOI:** 10.3390/vaccines14030275

**Published:** 2026-03-20

**Authors:** Nicolas D. Prather, Jadelynn Aki, Sean Jeffreys, Bernard P. Arulanandam, Chiung-Yu Hung, Jieh-Juen Yu

**Affiliations:** 1 Department of Molecular Microbiology and Immunology, University of Texas at San Antonio, San Antonio, TX 78249, USA; nicolas.prather@utsa.edu (N.D.P.); jadelynnlkaki@utexas.edu (J.A.); sean.jeffreys@swid-id.com (S.J.); chiungyu.hung@utsa.edu (C.-Y.H.); 2 Department of Immunology, Tufts University School of Medicine, Boston, MA 02111, USA; bernard.arulanandam@tufts.edu

**Keywords:** *Acinetobacter baumannii*, vaccine, multi-epitope vaccine, multidrug-resistant bacteria, nosocomial pathogen, reverse vaccinology

## Abstract

Background/Objectives: Multidrug-resistant (MDR) *Acinetobacter baumannii* (*Ab*) has emerged as a significant bacterial pathogen responsible for nosocomial infections. The most common clinical manifestations of *Ab* infection include ventilator-associated pneumonia and catheter-related bloodstream/urinary infections. Given the extensive MDR phenotype of *Ab*, preventive vaccination strategies are crucial for protecting susceptible populations. Methods: We utilized immunoinformatics to identify candidate peptides containing both putative B- and T-cell epitopes from proteins associated with *Ab* pathogenesis. Subsequently, we designed novel *Acinetobacter* Multi-Epitope Vaccines (AMEVs), each comprising an *Ab* thioredoxin A (TrxA) leader protein, five to seven of the identified peptide antigens, and a C-terminal His(6x)-tag to facilitate protein purification. Results: Subcutaneous vaccination of C57BL/6 mice with AMEV1 or AMEV2, formulated with TiterMax adjuvant, conferred 60% and 80% protection, respectively, against intraperitoneal *Ab* challenge. AMEV vaccination induced a robust antibody response to each corresponding whole protein and most of its component peptides. We then constructed an improved vaccine, AMEV5, which included the *Ab* TrxA protein and seven confirmed B-cell epitope peptides. Subcutaneous immunization of BALB/c mice (*n* = 10 per group) with rAMEV5 emulsified in Adda03 adjuvant activated antigen-specific IL-5-secreting T cells and antibody-producing B cells. Evaluation of vaccine efficacy demonstrated that AMEV2- and AMEV5-immunized mice were protected from a lethal intraperitoneal *Ab* challenge, with survival rates of 70% and 90%, respectively. Conclusions: These study results provide insights into the application of reverse vaccinology to combat the rise of MDR *Acinetobacter* infection.

## 1. Introduction

The *Acinetobacter baumannii* (*Ab*) is a multidrug-resistant (MDR) Gram-negative bacterium that is emerging as a major cause of hospital-acquired and combat-related infections. Patients at the highest risk of acquiring *Ab* infections include immunocompromised individuals and those hospitalized for extended durations, especially in intensive care units [1]. As a primarily nosocomial pathogen, *Ab* can cause pneumonia, skin and soft tissue infection, surgical site infection, burns, urinary tract infections, and device-related infections through devices like ventilators and catheters [2]. In recent years, drug-resistant strains of *Ab* have increased at an alarming rate due to their high genetic plasticity, highlighting the need for new antibiotics or treatment options [3,4]. Due to the pathogen’s environmental persistence, virulence, and limited treatment options, these infections result in high mortality rates and significant healthcare costs for the patients [5].

The extensive MDR and increasing prevalence of *Ab* underscore the urgent need to investigate and develop alternative strategies such as vaccines for use as immunotherapeutics to combat *Ab* infections. We have previously developed multi-epitope vaccines using reverse vaccinology [6]. Through this strategy, we selected 32 pathogenesis-associated proteins. Using EigenBio’s proprietary epitope prediction software, we identified 10 lead antigenic peptides from these proteins, each containing both B-cell and T-cell epitopes. These peptides were predicted to be surface exposed to ensure B-cell epitope accessibility, and to lack mouse and human homologs to limit autoimmune reactions. Previously, we reported the construction of a novel *Acinetobacter* Multi-Epitope Vaccine (AMEV2) using five of the 10 identified antigenic peptides [7]. We further demonstrated that vaccination with AMEV2 provided partial protection (60% survival) to mice against intranasal challenge with a virulent *Ab* clinical isolate. Here, we report the construction of AMEV1 using the remaining five of the 10 identified peptides. The protective efficacy of AMEV1 and AMEV2 vaccines against *Acinetobacter* sepsis was evaluated. The antigenicity of component peptides was assessed to construct a refined vaccine, designated AMEV5. The AMEV5 vaccine’s immunity and protective efficacy were investigated and are reported here using a mouse model of *Acinetobacter* sepsis.

## 2. Materials and Methods

### 2.1. Animals

Animal experiments were performed using 7–8-week-old female C57BL/6 mice purchased from Jackson Laboratories (Bar Harbor, ME, USA) or BALB/c mice from Charles River Laboratories (Wilmington, MA, USA). All animal experiments were conducted in accordance with Institutional Animal Care and Use Committee Protocol MU070 in an AAALAC-accredited animal facility at the University of Texas at San Antonio.

### 2.2. Bacterial Culture

*Ab* AB5075 isolate was obtained from The University of Washington (Seattle, WA, USA) [8]. All laboratory consumables were purchased from Fisher Scientific (Waltham, MA, USA) unless specified. The bacterium was streak-plated on Luria–Bertani (LB) agar plates supplemented with ampicillin (100 µg/mL). An overnight liquid culture was prepared by inoculating ampicillin-supplemented LB medium with a single bacterial colony and incubated in a 37 °C shaking incubator. The next day, the culture was subcultured to an OD600 nm = 0.03 in fresh LB broth without antibiotics and grown to mid-log phase (~3.5 h). The bacteria were then collected by centrifugation, washed twice in phosphate-buffered saline (PBS), and diluted to an OD600 nm = 0.5 to achieve a concentration of about 2 × 10^8^ CFUs/mL. This inoculum was further diluted to create the intraperitoneal challenge dose, which was later determined by serial dilution plating.

### 2.3. rAMEVs Cloning and Purification

rAMEV1 and rAMEV5 encoding nucleotide sequences with optimized codons for *E. coli* expression were synthesized and cloned into the pET-23a (+) plasmid (GenScript, Piscataway, NJ, USA). The AMEVs expression vectors were used to transform *E. coli* BL21(DE3) for recombinant protein production. The rAMEV1 and rAMEV5 were purified to high homogeneity with cobalt affinity chromatography using the HisPur Cobalt Purification Kit (Thermo Fisher, Waltham, MA, USA) according to the manufacturer’s recommendation. The purified protein identity was confirmed by subjecting protein trypsin digests to liquid chromatography tandem mass spectrometry (LC-MS/MS) analysis at the University of Texas at San Antonio Mass Spectrometry & Proteomics Core. Generation of rAMEV2 was reported previously [6].

### 2.4. Vaccination and Challenge

Mice (*n* = 10 per group) were subcutaneously vaccinated with 100 µL of rAMEVs (10 µg) or PBS (mock) with adjuvant at a 1:1 (*v*/*v*) ratio on days 0, 14, and 28. The adjuvant used in the vaccination study was either TiterMax Gold (Sigma-Aldrich, St. Louis, MO, USA) or AddaS03 adjuvant (InvivoGen, San Diego, CA, USA) as indicated. After the final vaccination, the mice were allowed to rest for 4–5 weeks before being intraperitoneally challenged with a lethal dose of *Ab* (~3 × 10^6^ CFUs /100 µL/mouse) to evaluate protective efficacy by monitoring the survival rate.

### 2.5. Determination of Antibody Levels by ELISA

Antiserum reactivity with rAMEVs and their component peptides was measured as previously reported [6]. Briefly, for whole protein titers, microtiter plates (Corning #9018, Glendale, AZ, USA) were coated overnight with rAMEVs (500 ng/well) in 0.1 M sodium carbonate buffer (pH 9.5). Serially 2-fold diluted sera (starting at 1:100) were used as primary antibodies. Sample wells without mouse sera were used as blank controls. A peroxidase-labeled goat anti-mouse Ig(H+L) antibody (1:4000 dilution; #1010-05 SouthernBiotech, Birmingham, AL, USA), goat anti-mouse IgG1 (1:5000, #1070-05), and goat anti-mouse IgG2a (1:5000 dilution, #1080-05) served as the secondary antibody in the indirect ELISA assay. TMB substrate reagent (BD Biosciences, San Diego, CA, USA) was added to each well for color development. Reactions were stopped by adding 2 M H_2_SO_4_ and the absorbance at 450 nm was recorded using a microplate reader (TECAN, Männedorf, Switzerland). Endpoint titers with a 95% confidence level were determined using a previously reported statistically defined method [9]. For reactivity with component peptides, the ELISA assay was conducted as described above, except microplate wells were coated with individual component peptide (1 µM) and reacted with 1:500 diluted mouse sera.

### 2.6. T-Cell ELISpot Assays

The frequency of IL-5 secreting cells was determined using a T-cell ELISpot assay as previously described [6]. Briefly, mice were vaccinated twice, 2 weeks apart, and spleens were collected 7 days after the booster vaccination. PVDF membrane ELISpot plates (Millipore Sigma, Burlington, MA, USA) were coated with IL-5 capture antibody (clone: TRFK5, 5 µg/mL). Splenocytes (5 × 10^5^ per well) were stimulated with either hen egg lysozyme (HEL) as a negative control, αCD3 as a positive control, or rAMEV5. An unstimulated PBS-only well was also prepared as a basal level control. Biotinylated IL-5 monoclonal antibody (clone: TRFK4, 0.5 µg/mL) was used as the detection antibody. Streptavidin Alkaline Phosphatase (AP) conjugate (Invitrogen, Waltham, MA, USA) and BCIP/NBT phosphatase substrate (SeraCare, Milford, MA, USA) were used for spot color development. Spots were counted using an ImmunoSpot analyzer (Cellular Technology Limited, Cleveland, OH, USA).

### 2.7. B-Cell ELISpot Assays

The frequency of antibody-secreting cells was determined using a B-cell ELISpot assay, as previously described with minor modifications [6]. Briefly, PVDF membrane ELISpot plates were coated overnight with 1 µg per well of either HEL (negative control), goat anti-mouse Ig (SouthernBiotech, positive control), or rAMEV5. PBS-only wells served as an additional negative control. Splenocytes were seeded at a density of 2.5 × 10^5^ per well and incubated for 6 h at 37 °C with 5% CO_2_. AP-conjugated goat anti-mouse-Ig secondary antibodies and BCIP/NBT phosphatase substrate were used to detect spots.

### 2.8. Statistical Analysis

Statistical analyses were conducted using Prism 10 (GraphPad Software, Boston, MA, USA). Differences between mock and AMEV-vaccinated groups were assessed using Student’s *t*-test. Survival rates were analyzed with the Log-rank Mantel–Cox test. Differences were considered statistically significant when *p* ≤ 0.05.

## 3. Results

### 3.1. Generation and Evaluation of Acinetobacter Multi-Epitope Vaccines, AMEV1 and AMEV2

**Figure 1:**
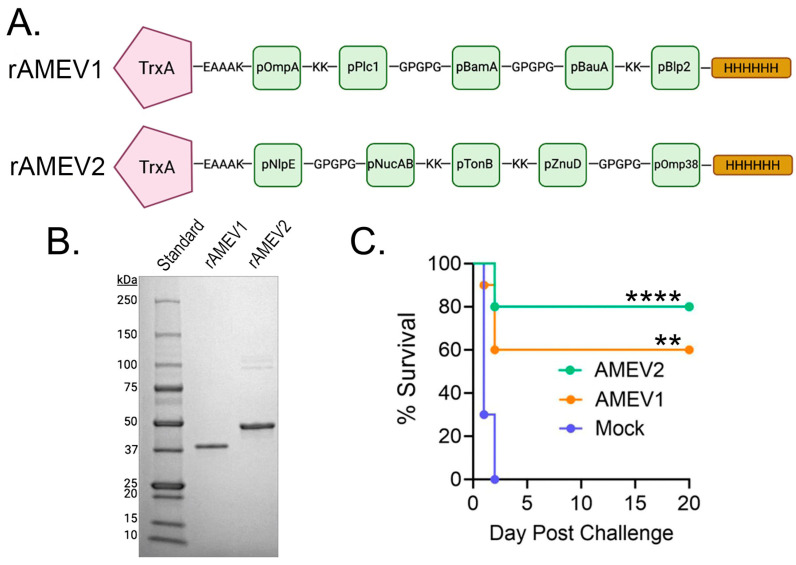
Evaluation of AMEV1 and AMEV2 vaccines against *Acinetobacter* sepsis. (**A**) Diagram of rAMEV1 and rAMEV2 consisting of *Ab* thioredoxin linked to five predicted immunogenic peptides, followed by a six-histidine tag. The thioredoxin leader protein is linked to the first peptide by a rigid EAAAK linker, while the peptides are linked by KK and GPGPG linkers to ensure epitope separation. (**B**) 1 µg of metal-affinity purified rAMEV1 (predicted molecular mass 38.3 kDa) and rAMEV2 (41.5 kDa) proteins were separated on 12% SDS-PAGE and stained with Coomassie Brilliant Blue G-250. (**C**) C57BL/6 mice (*n* = 10) were subcutaneously vaccinated three times with AMEV1, AMEV2, or PBS (mock, TiterMax Gold adjuvant) at two-week intervals and then rested for nearly 5 weeks following the second boost. Mice were challenged with 2.07 × 10^6^ CFUs of *Ab* AB5075 strain on day 60. Survival of mice was monitored for 20 days. ** *p* = 0.0010, **** *p* < 0.0001, Log-rank (Mantel–Cox) test compared to mock group.

We previously reported the development of a reverse vaccinology pipeline for selecting *Ab* peptide antigens from virulence factors crucial for bacterial pathogenesis [6]. A total of 10 antigenic peptides (Table 1), each containing putative B- and T-cell epitopes were selected for constructing AMEVs. These identified peptides ranging from 33 to 67 amino acids were conserved among *Ab* strains, suggesting potential for broad treatment application. Two subunit vaccines, AMEV1 and AMEV2, were constructed (Figure 1A). Each vaccine consisted of *Ab* thioredoxin A (GenBank: AKA32972.1), a known virulence factor [10,11], linked via a rigid EAAAK linker to five of the identified peptides. These peptides were connected to each other by either KK or GPGPG linkers. Additionally, the protein included a terminal 6-histidine tag to aid in protein purification. Both proteins were purified to high purity (Figure 1B), and their identity was confirmed by LC-MS/MS proteomic analysis. The rAMEV1 and rAMEV2 were formulated with TiterMax Gold adjuvant to enhance antibody production, which is critical for protection against *Ab* infection. C57BL/6 mice that received three doses of AMEV1 and AMEV2 vaccines subcutaneously generated robust vaccine-specific antibody responses, showing endpoint titers greater than 1:1 × 10^5^ dilution for all immunized mice 3 days prior to *Ab* challenge. Both AMEV1 and AMEV2 vaccines were efficacious and provided partial protection against hypervirulent AB5075 systemic infection via intraperitoneal injection. As shown in Figure 1C, all mock (PBS plus adjuvant) vaccinated mice succumbed to the acute infection from 2.07 × 10^6^ CFUs (~4 × LD50) within two days, while AMEV1 and AMEV2 vaccination achieved 60% and 80% survival rates, respectively.

### 3.2. Assessment of Component Peptide Immunogenicity for rAMEV1 and rAMEV2

**Figure 2:**
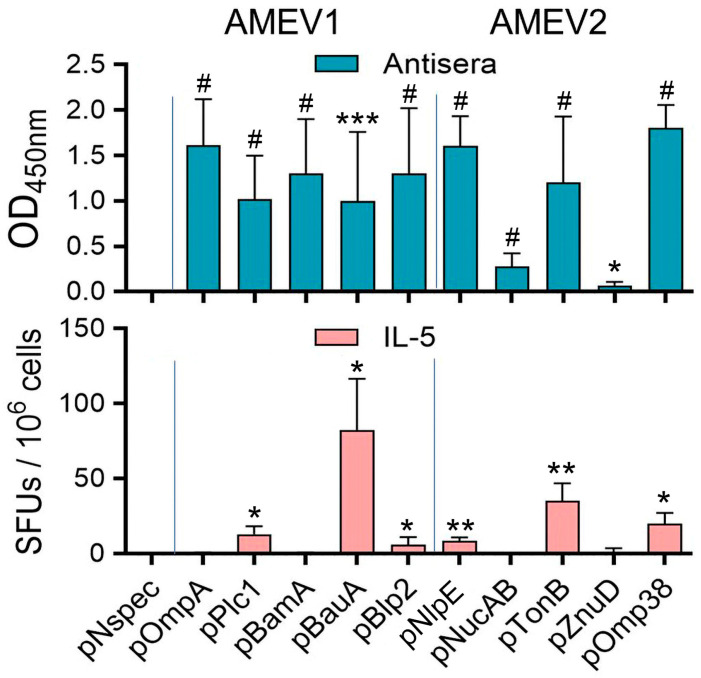
Assessment of AMEV1 and AMEV2 component peptide immunogenicity. C57BL/6 mice were vaccinated subcutaneously with TiterMax Gold adjuvanted rAMEV1 (10 µg), rAMEV2 (10 µg), or PBS (mock) on days 0, 14, and 28. On day 53, sera (*n* = 10 per group) were collected and diluted 1:500 to measure antibody reactivity with corresponding AMEV component peptides by ELISA. Splenocytes (*n* = 3 per group) were processed to determine the numbers of IL-5 secreting immune cells by stimulation with 1 µM of each indicated peptide in an ELISpot analysis. *Coccidioides posadasii* BGL4, a 33-amino-acid-long peptide (pNspec) from an unrelated fungal antigen, was used as a nonspecific control. Data are presented as mean ± SD, * *p* < 0.05, ** *p* < 0.01, *** *p* < 0.001, ^#^ *p* < 0.0001, unpaired t-test compared to pNspec.

To verify the immunogenicity of the 10 bioinformatics-identified AMEV component peptides, we collected immune sera and splenocytes from vaccinated mice. We assessed peptide-specific antibody reactivity by ELISA and Th2 cell activation by ELISpot, respectively. As shown in Figure 2 upper panel, AMEV1-vaccinated mice produced antibodies against all its component peptides (pOmpA, pPlc1, pBamA, pBauA, and pBlp2), while AMEV2-vaccinated mice generated anti- pNlpE, pNucAB, pTonB, and pOmp38 antibodies. These serum reactivity data indicate that at least nine identified peptides are antigens containing B-cell epitopes. IL-5 is known as a key regulator for eosinophil development and activation; however, it also plays an important role in B-cell growth and enhancement of immunoglobulin secretion [12,13]. Vaccination of mice with AMEV1-generated Th2 clones that secreted IL-5 upon stimulation with pPlc1, pBauA, and pBlp2 peptides (Figure 2, lower panel). Similar ELISpot assays revealed that rAMEV2 component peptides, pNlpE, pTonB, and pOmp38 are T-cell epitope antigens (Figure 2, lower panel).

### 3.3. Construction of AMEV5

**Figure 3:**
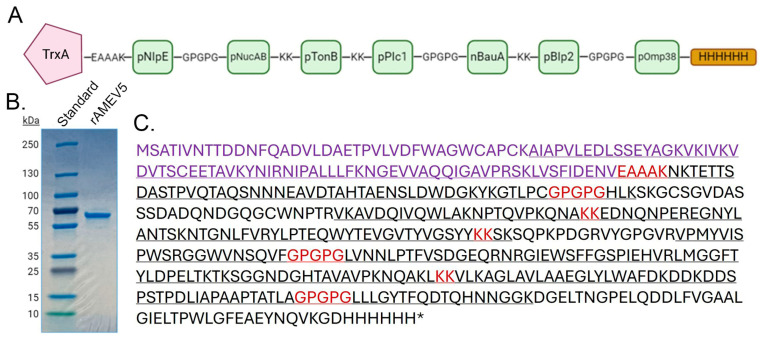
Generation of a multi-epitope vaccine AMEV5. (**A**) Diagram of rAMEV5 comprising *Ab* thioredoxin A (TrxA) linked to seven selected peptides and a C-terminus His-tag. (**B**) SDS-PAGE analysis of the rAMEV5 purified by immobilized cobalt-affinity chromatography. (**C**) Amino acid sequence of rAMEV5 with matched peptides identified by LC-MS/MS in underline (73% protein sequence coverage). TrxA protein sequence is depicted in purple, and linkers connecting antigenic peptides are shown in red.

Following the antigenicity characterization of the 10 identified peptides, we constructed the AMEV5 vaccine aiming to improve protective efficacy against *Ab* infection. We used AMEV2 as the backbone for the new vaccine construct because it was better characterized [6,7] and provided superior protection compared to AMEV1 (Figure 1C). Specifically, we removed AMEV2 pZnuD which showed minimal antibody reactivity and Th2 (IL-5) activation, and added three AMEV1-confirmed antigenic peptides, pPlc1, pBauA and pBlp2, to form AMEV5 (Figure 3A). The recombinant AMEV5 (490 aa) was expressed in *E. coli* and purified to high homogeneity (Figure 3B). The identity of the purified rAMEV5 was confirmed by LC-MS-MS protein sequencing (Figure 3C).

### 3.4. Evaluation of AMEV5

**Figure 4:**
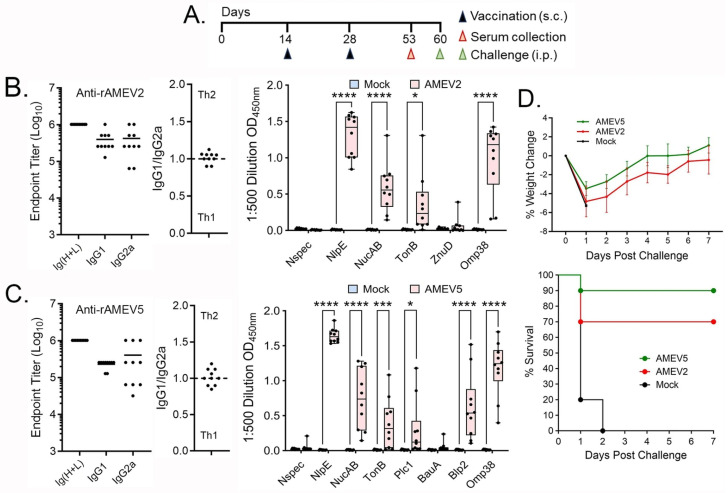
Evaluation of the AMEV5 vaccine. (**A**) Seven- to eight-week-old BALB/c mice (*n* = 10 per group) were subcutaneously vaccinated with AddaS03-adjuvanted rAMEV2 (10 µg), rAMEV5 (10 µg), or PBS (mock) on days 0, 14, and 28. On day 53 (week 8), sera were collected to determine anti-rAMEV2 (**B**) or anti-rAMEV5 (**C**) whole protein total Ig, IgG1 and IgG2a titers, IgG1/IgG2a ratio, and component peptide reactivity (box and whiskers plot showing minimum, maximum, median, and the 25th and 75th percentile). (**D**) Mice were rested for 4 weeks after the final vaccination before intraperitoneal (i.p.) challenge with a lethal dose (4.25 × 10^6^ CFU) of AB5075 isolate. Mice were monitored for morbidity (weight loss) and mortality (survival) for 7 days. * *p* < 0.05, *** *p* < 0.001, **** *p* < 0.0001, unpaired *t*-test.

AMEV5-mediated humoral responses and vaccine efficacy were assessed with the AMEV2 vaccine used for comparison. BALB/c mice were vaccinated subcutaneously with the same dose of rAMEV2 (10 μg), rAMEV5 (10 μg), or PBS formulated with AddaS03 (adjuvant) on days 0, 14, and 28 (Figure 4A). Sera were collected at week 8 post-priming to determine anti-rAMEV2 and anti-rAMEV5 total Ig, IgG1 and IgG2a endpoint titers. Additionally, sera were diluted 1:500 to assess reactivity with each component peptide. As shown in Figure 4B, AMEV2 vaccination induced robust anti-rAMEV2 antibody production prior to challenge (week 8). Isotype analyses revealed the production of both Th1- (IgG2a) and Th2- (IgG1) type antibodies, suggesting that AMEV2 vaccination likely induces a balanced Th1 and Th2 immunity, as indicated by IgG1/IgG2a (Log10 titer) ratios. AMEV2 antisera reacted with most of the rAMEV2 component peptides except pZnuD (Figure 4B), similar to observations in vaccinated C57BL/6 mice (Figure 2 upper panel). Similarly, AMEV5 vaccination induced high titers of antibodies against the rAMEV5 whole protein and most of its component peptides except pBauA (Figure 4C). A balanced Th1 and Th2 immunity was induced by AMEV5 vaccination (Figure 4C). We further evaluated vaccine protective efficacy against *Acinetobacter* septicemia. All AMEV2- and AMEV5-vaccinated mice and adjuvant-alone control mice were challenged 4 weeks post-final boost with a lethal dose of the hypervirulent AB5075 strain via the intraperitoneal route. Due to the acute nature of the infection in the mouse model, all AB5075-infected animals began losing weight 1 day after challenge, then started to recover if they did not succumb to infection (Figure 4D, upper panel). Overall, although not statistically significant, the AMEV5-vaccinated mice showed less weight loss and a higher survival rate (90%) compared to AMEV2-immunized mice (70%) (Figure 4D, lower panel). All unvaccinated mice succumbed to infection within 2 days.

### 3.5. AMEV5 Vaccination Generated Adaptive Immune Memory

**Figure 5:**
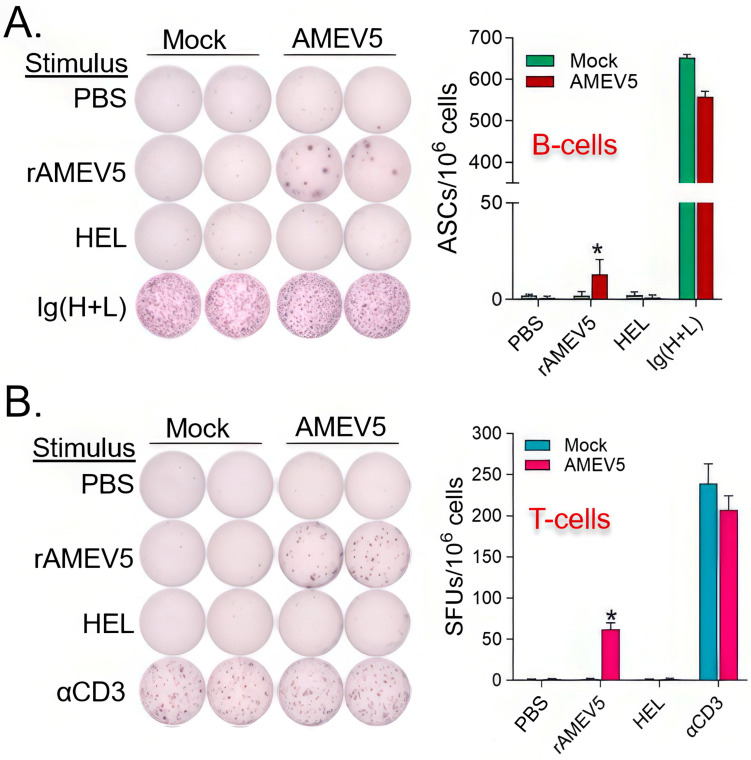
AMEV5 vaccination generated rAMEV5-specific antibody-producing B-cells and IL-5 secreting T-cells in the spleen. BALB/c mice (*n* = 3 per group) were subcutaneously vaccinated with either PBS + AddaS03 (mock) or rAMEV5 (10 µg) + AddaS03 (AMEV5) on days 0 and 14. Splenocytes were prepared on day 21. (**A**) Representative B-cell ELISpots are shown, and the frequency of antibody secreting cells (ASCs) was calculated for cells that secreted antibodies reacting with PBS, rAMEV5 or hen egg lysozyme (HEL, an unrelated antigen control). Wells coated with goat anti-mouse Ig(H+L) served as positive control. (**B**) Representative T-cell ELISpots are shown, and spot forming units (SFUs) were calculated for cells that secreted IL-5 upon stimulation with PBS, rAMEV5, or HEL. Splenocytes stimulated with α-CD3 were used as a positive control. Data are presented as mean ± SD. * *p* < 0.05, unpaired t-test compared to mock.

The activation of memory adaptive immunity is important for effective vaccines. We collected spleens from PBS mock and AMEV5-vaccinated mice (*n* = 3 per group) one week post a booster shot. Pooled splenocytes were used to determine B-cells that secreted antibodies reacting with PBS, hen egg lysozyme (HEL, an unrelated control), or rAMEV5 by ELISpot analysis. Wells coated with goat anti-mouse Ig(H+L) served as positive control. Pooled splenocytes were also stimulated with media, rAMEV5, HEL, and αCD3 (positive control) to detect IL-5-secreting T cells. The results of the ELISpot assessment indicated that AMEV5 vaccination generated anti-rAMEV5 producing B-cells (Figure 5A) and activated rAMEV5 antigen-specific Th2 cell clones (Figure 5B).

## 4. Discussion

Vaccine development against *Acinetobacter* infection is in its early stages. In 2010, McConnell and Pachon [14] reported the first experimental *Acinetobacter* vaccine results using a formalin-inactivated whole-cell vaccine and provided evidence that active and passive vaccination effectively controlled *Acinetobacter* infection in a murine model of disseminated sepsis. Since then, several laboratories have begun developing *Ab* vaccines based on inactivated whole cells as well as subunit antigens [15,16,17,18,19,20,21]. In general, whole-cell vaccines have provided better protection against *Ab* infection compared to subunit vaccines [22]. However, subunit vaccines are considered a safer alternative. Recent articles by Lau and Tan [23] and Yang et al. [24] reviewed the development of subunit *Acinetobacter* vaccines, including methodologies for new antigen candidate discovery, the efficacy of reported pre-clinical subunit vaccines, animal models, and challenges to clinical translation. Among reported subunit vaccines, in silico B-cell and T-cell epitope prediction has been applied to construct multiepitope vaccines. For example, Du et al. designed an rOmp22 multiepitope vaccine containing two T-cell epitopes and three B-cell epitopes from a single protein [25]. Similarly, another multiepitope vaccine was constructed by Ren et al. using T-cell and B-cell epitopes from each of the two outer membrane proteins, FilF and NucAb, fused to an Ata protein backbone [26]. Both vaccines conferred protection against *Ab* infection. In contrast to the two previously described multiepitope vaccines that used separate B-cell or T-cell antigens, we screened for peptides containing both epitopes to boost Th2-mediated B-cell activation and antibody production [6]. We applied immunoinformatics analysis to identify 10 antigenic peptides from proteins associated with *Acinetobacter* pathogenesis for vaccine development. We selected highly conserved epitopes from multiple virulence factors, allowing the AMEVs to simultaneously target multiple critical mechanisms of infection and provide broad protection against various clinical isolates. The proteins we identified are predicted to be localized on the surface of the bacterial outer membrane. BamA has been shown to play a role in the assembly and insertion of β-barrel proteins into *E. coli* outer membranes [27]. Active and passive immunization with AbBamA protected mice against systemic MDR *Ab* infections, resulting in survival rates of 80% and 60%, respectively [28]. BauA is a siderophore that aids in iron acquisition [29]. Blp2 contains an Ig-like repeat that assists with biofilm formation [30]. NlpE is a copper resistance lipoprotein that also helps with adhesion [31]. NucAB is a part of the NucH family of extracellular endonucleases that prevent phages from infecting bacteria [32]. Omp38 is a protein that induces apoptosis of epithelial cells at the beginning of infection [33]. OmpA is involved in various biological processes, including the regulation of invasion, biofilm formation, and immune response [34,35]. Targeting OmpA as a therapeutic for *Ab* infection has been proposed [36]. Subunit OmpA vaccines have also been evaluated, demonstrating 50% and 90% protective efficacy following active and passive vaccination, respectively [21]. The genome annotated TonB is a putative TonB-dependent metal transporter that might facilitate iron acquisition and bacterial growth [37]. Plc1 is a phospholipase that presumably interferes with the host’s inflammatory response to infection [38,39]. The antigenicity of these immunoinformatic-identified peptides was experimentally validated using the murine model of *Acinetobacter* infection.

Adjuvants are critical components of vaccine formulations that enhance and shape antigen-specific immune responses. TiterMax was developed as an alternative to complete Freund’s adjuvant for producing antisera in animals and has been shown to induce higher, longer-lasting titers [40]. We used the improved TiterMax Gold adjuvant which contains a copolymer (CRL-8300), squalene (a metabolizable oil), and a sorbitan monooleate for the initial AMEV study to ensure robust antibody production. This allowed us to evaluate AMEV1- and AMEV2-induced immunity and protective efficacy. For clinical relevance, we switched to AS03 adjuvant in the later AMEV2 and AMEV5 formulations. AS03-adjuvanted H1N1 influenza vaccines have been approved by the European Medicines Agency (EMA) and the AS03-adjuvanted monovalent avian influenza A (H5N1) vaccine was approved by FDA for stockpile [41,42]. AS03 (GlaxoSmithKline [43]; AddaS03, InvivoGen), like TiterMax, is a squalene-based emulsion adjuvant that generates strong B-cell responses and antibody production by inducing a strong innate immune response to enhance quantitative and qualitative antigen presentation [44]. Comparable antibody production was observed in C57BL6 mice vaccinated with rAMEV2 + TiterMax Gold and BALB/c mice immunized with rAMEV2 + AddS03. These data validate the antigenicity of the AMEV2 component peptide in two different MHC-II strains with potent Th2 adjuvants indicating plausible promiscuous vaccine antigen presentation. Previously, we reported AMEV2-mediated protection against pulmonary challenge with clinical *Ab* isolate Ci79 [6]. Here, we demonstrated its protection against *Ab* AB5975 systemic infection suggesting a broad protection against MDR *Acinetobacter* through AMEV2 vaccination.

Despite the presence of pBauA in rAMEV5, confirmed by LC/MS/MS analysis (Figure 3C) and showing relatively high reactivity with AMEV1 antisera, AMEV5 vaccination did not generate a good titer of anti-pBauA antibody. When multiple epitopes are presented together, the immune system rarely responds to all of them equally, as shown in Figure 1 and Figure 4. This antigenic competition and immunodominance may occur because highly immunogenic peptides outcompete others for binding to MHC molecules or B-cell receptors [45]. Furthermore, alterations of epitope immunodominance by adjuvants in vaccine formulation have been reported [46]. It is possible that the different adjuvants used for AMEV1 (TiterMax Gold) and AMEV5 (AddaS03) contribute to the difference in pBauA immunogenicity; however, a head-to-head comparison of AMEV5 formulated with both adjuvants would be required to confirm this. Additionally, in the rAMEV5 construct, relocating pBauA next to pPlc1 may have induced a conformational change, potentially hindering B-cell receptor access to the epitope compared to the rAMEV1 construct. Therefore, the cause of low pBauA immunogenicity following AMEV5 vaccination, including epitope conformation, antigen presentation, and dominance of other epitopes, remains to be fully elucidated. Nevertheless, the rAMEV5 construct contains B-cell epitopes from multiple virulence factors that could generate antibodies to inhibit bacterial adhesion and nutritional uptake. These antibodies could also contribute to opsonophagocytic killing of *Ab* via complement activation and Fc-receptor binding [7,47,48].

While our pre-clinical AMEVs show promise, several limitations and concerns exist. First, the mouse model of *Ab* infection used in this study, though well-accepted, is highly acute, with unprotected animals succumbing to infection within 48 h. Therefore, it is questionable whether vaccine-induced adaptive memory can be activated rapidly enough to significantly contribute to protection against *Ab* infection. Recently, Dr. Feldam’s group established a chronic *Ab* pneumonic infection mouse model using *tlr4* mutant mice [49]. However, the suitability of this chronic model for vaccine development remains to be determined, as live attenuated vaccines and some subunit adjuvants may require TLR4 signaling for effective priming. Second, although we and others have demonstrated the protective capacity of vaccine-induced antibodies, vaccine durability remains to be evaluated for AMEVs [7,14,50,,51]. Third, clinical *Ab* vaccine administration would most likely a targeted approach rather than universal immunization. The primary target population would consist of high-risk individuals, such as patients anticipating prolonged intensive care unit admissions, those requiring mechanical ventilation or central venous catheters, severe burn victims, and military personnel deployed to high-risk trauma zones. Therefore, the development of such a nosocomial vaccine faces significant economic barriers, as the relatively small and specific target population limits the traditional commercial return on investment for pharmaceutical companies. Nevertheless, studying the AMEVs allows us to identify potential protective antigens that can be further developed as therapeutic monoclonal antibodies for clinical *Ab* treatment.

## 5. Conclusions

As MDR *Ab* increasingly evades traditional antibiotic therapies, prophylactic strategies are urgently needed to protect vulnerable patient populations in hospital settings. Using reverse vaccinology to rationally select and combine highly conserved immunogenic epitopes, we successfully generated novel multi-epitope vaccines targeting MDR *Ab*. We validated the antigenicity of the predicted epitopes and their derived AMEVs using a murine model. The successful induction of antigen-specific memory B-cells and IL-5-secreting Th2 cells across different murine MHC-II backgrounds (C57BL/6 and BALB/c) underscores the broad immunogenicity of these constructs. Because sepsis and pneumonia are leading causes of death from *Ab* infection, we demonstrated that the refined AMEV5 conferred protection against a lethal, hypervirulent *Ab* systemic challenge. Future studies will determine the protective efficacy of AMEV5 against pulmonary infections by additional clinical isolates. These findings validate this multi-epitope approach as a highly promising and scalable platform. Overall, our findings provide insights for further research and development of multi-epitope vaccines that can enhance immune responses across diverse populations in the rise of MDR *Ab*. Additionally, our findings pave the way for developing AMEV-based monoclonal antibodies that can be used as alternative or adjunctive therapeutics with antibiotics to treat MDR *Ab* infection. Ultimately, these results establish an essential foundation for clinical translation, offering a robust new strategy to combat a critical public health threat.

## Figures and Tables

**Table 1:** Predicted immunogenic peptides by immunoinformatic that were selected for AMEVs construction.

Peptide Antigen	Derived from Protein Name	NCBI Reference Sequence	Peptide Amino Acid Sequence
pOmpA	OmpA family protein	WP_000026486.1	TSSTAPPLAAATETTGKSRGFLPIIALIILGLL
pPlc1	Phosphatidylcholine-specific phospholipase C	WP_001081748.1	SKSQPKPDGRVYGPGVRVPMYVISPWSRGGWVNSQVF
pBamA	outer membrane protein assembly factor (Oma87)	WP_000171057.1	NLQETKQNDSSPEEVGGNALVQFGTELVLPMPFKGDWTRQVRP
pBauA	TonB-dependent ferric acinetobactin receptor BauA	WP_001016286.1	LVNNLPTFVSDGEQRNRGIEWSFFGSPIEHVRLMGGFTYLDPELTKTKSGGNDGHTAVAVPKNQAKL
pBlp2	Ig-like repeat protein Blp2	WP_000196831.1	VLKAGLAVLAAEGLYLWAFDKDDKDDSPSTPDLIAPAAPTATLA
pNlpE	copper resistance protein NlpE	WP_000749178.1	NKTETTSDASTPVQTAQSNNNEAVDTAHTAENSLDWDGKYKGTLPC
pNucAB	ExeM/NucH family extracellular endonuclease	WP_000847239.1	HLKSKGCSGVDASSSDADQNDGQGCWNPTRVKAVDQIVQWLAKNPTQVPKQNA
pTonB	TonB-dependent siderophore receptor	WP_001998816.1	EDNQNPEREGNYLANTSKNTGNLFVRYLPTEQWYTEVGVTYVGSYY
pZnuD	zinc piracy TonB-dependent receptor ZnuD	WP_000899872.1	LSKEKSNNVELGLHFDNDKLDYHLHVYHNWFDDYIYAQTLDR
pOmp38	OmpA family protein	WP_000777885.1	LLLGYTFQDTQHNNGGKDGELTNGPELQDDLFVGAALGIELTPWLGFEAEYNQVKGD

## Data Availability

Data are contained within the article.
